# D-Amino Acids as a Biomarker in Schizophrenia

**DOI:** 10.3390/diseases10010009

**Published:** 2022-01-31

**Authors:** Kurumi Taniguchi, Haruka Sawamura, Yuka Ikeda, Ai Tsuji, Yasuko Kitagishi, Satoru Matsuda

**Affiliations:** Department of Food Science and Nutrition, Nara Women’s University, Kita-Uoya Nishimachi, Nara 630-8506, Japan; kn9@gmail.com (K.T.); swmuuu55@icloud.com (H.S.); tyvufkxaq1226-218@outlook.jp (Y.I.); ai.tsuji0225@gmail.com (A.T.); y_kitagishi@live.jp (Y.K.)

**Keywords:** schizophrenia, D-Serine, D-Aspartate, gut microbiota, racemase

## Abstract

D-amino acids may play key roles for specific physiological functions in different organs including the brain. Importantly, D-amino acids have been detected in several neurological disorders such as schizophrenia, amyotrophic lateral sclerosis, and age-related disorders, reflecting the disease conditions. Relationships between D-amino acids and neurophysiology may involve the significant contribution of D-Serine or D-Aspartate to the synaptic function, including neurotransmission and synaptic plasticity. Gut-microbiota could play important roles in the brain-function, since bacteria in the gut provide a significant contribution to the host pool of D-amino acids. In addition, the alteration of the composition of the gut microbiota might lead to schizophrenia. Furthermore, D-amino acids are known as a physiologically active substance, constituting useful biomarkers of several brain disorders including schizophrenia. In this review, we wish to provide an outline of the roles of D-amino acids in brain health and neuropsychiatric disorders with a focus on schizophrenia, which may shed light on some of the superior diagnoses and/or treatments of schizophrenia.

## 1. Introduction

Racemization of L-amino acids reacted by several racemases could lead to the synthesis of D-amino acids. Amino acids are usually found in these two forms in nature, with the exception of glycine, which has no chiral center. The amino acid racemases have been broadly identified in humans, mice, invertebrate animals, and bacterial species [1]. In humans, D-amino acid studies have recognized the relative abundance of D-amino acids in the brain as much as in several body fluids together with blood plasma, urine, and/or cerebrospinal fluid (CSF) [2,3]. Furthermore, D-Serine has been suggested as a possible biomarker for Alzheimer’s disease [3]. Additionally, several D-amino acids are detected in lactic fermentation foods [4]. Consistently, various studies have also revealed the presence of D-amino acids within several types of fermented foods and drinks [5]. It has been indicated that some D-amino acids within vinegar are commonly produced from lactic acid bacteria [4,6]. Furthermore, D-amino acids including D-Tryptophan, D-Phenylalanine, D-Serine, and/or D-Alanine seem to have a sense of taste that is syrupier than sugar [6,7]. In general, a lot of D-amino acids may taste sweet when compared to the corresponding L-amino acids [7]. Food processing such as alkali treatment and/or long period heating is a well-known procedure to provide the racemization of L-amino acids [8]. The main quantity of D-amino acids in a body may usually result from the food intake and/or gut microbial synthesis [8,9]. Hence, gut microbiota could be important contributors to the production of systemic D-amino acids [9]. In the gut microbiota, D-amino acid production occurs via intrinsic amino acid racemases of the specific bacteria [10]. Among them, broad-spectrum amino acid racemases have been detected within certain gram-negative bacteria [11]. These racemases might have an impact on the microbial ecology [11]. Physiologically, D-amino acids have been suggested to control cell wall biogenesis, biofilm degradation, and/or spore germination in the microbiota [12,13]. 

Recently, D-amino acids have been known as signaling molecules in cells in order to keep embryonic neural homeostasis in the developing brain [14]. In addition, D-amino acids are correlated with brain and/or neurological disorders [15]. D-amino acids seem to have been recognized as essential signaling molecules in the central nervous system (CNS). In this review, we would like to go over the roles of D-amino acids in brain health and/or neuropsychiatric disorders with a focus on schizophrenia. Schizophrenia is a severe neuropsychiatric disorder, and its etiology remains basically unknown, which results nowadays in significant socioeconomic burdens [16]. Several environmental and/or genetic factors have been reported to play key roles in the pathogenesis of schizophrenia [17]. 

## 2. D-Amino Acids in Brain

**Figure 1:**
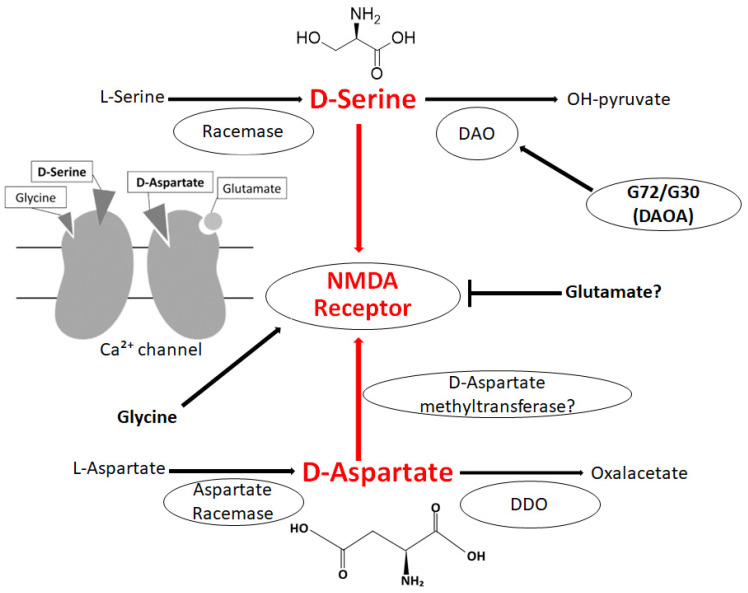
Illustration of the general D-Serine or D-Aspartate metabolic pathway in bacteria and/or in mammals. D-Amino acid oxidase (DAO) catalyzes the oxidative deamination of D-Serine. The DAO activator G72/G30 could stimulate the DAO. Both D-Serine and D-Aspartate are involved in NMDA receptor signaling in the neuron. The arrowhead means stimulation and/or augmentation, whereas the hammerhead represents inhibition. As a footnote, some serious events have been omitted for simplicity. DDO: D-Aspartate oxidase; DAOA: DAO activator; NMDA: N-methyl-D-aspartate.

The *N*-methyl-D-aspartate (NMDA)-type glutamate receptor is involved in the synaptic function of neurons. D-amino acids are agonists or co-agonists of the NMDA receptor, which is therefore crucial in synaptic plasticity [18,19]. Activating the NMDA receptor by D-amino acids might be essential for the positive sensitization of neuron, suggesting a key role for calcium ion-influx via the NMDA receptor for synaptic plasticity. In this regard, recognized functions of D-amino acids might embrace neurotransmission, synaptic plasticity, learning, and memory through modulating the NMDA receptors in the brain [20,21]. In fact, both D-Serine and D-Aspartate are involved in several processes underlying the NMDA receptor activation and neurotransmission in the CNS [22,23]. Furthermore, the absence of D-Serine is one of the mechanisms underlying the decrease of long-term potentiation and cognition [23]. D-amino acids exist predominantly in the frontal areas of the brain [24,25]. High amounts of D-Serine are also identified in the hippocampus and hypothalamus [25,26]. D-Serine may function as activating the NMDA receptor at the glycine binding site and may play a critical role in synaptic plasticity [27] (Figure 1). Consistent with this, several studies have revealed that D-Serine is an endogenous ligand for the NMDA receptor and is crucial in human neurophysiology, which might serve as a basis for pharmacological applications in D-Serine therapy [28]. In general, the metabolism of D-Serine is determined by the activity of racemases and/or D-amino acids oxidase (DAO). In mammals, the DAO is predominantly expressed in the CNS and/or in the cytosol of neurons [29], which is responsible for the metabolism of D-Serine, and has been implicated in the pathogenesis of neuropsychiatric diseases [29,30]. Similarly, it has been suggested that the DAO is involved in the regulation of neurotransmission in the CNS [30]. Serine racemase deficiency may induce a disturbed NMDA receptor related to synaptic neuroplasticity [31]. Therefore, a deleted or decreased Serine-racemase expression may also be associated with cognitive disorders such as schizophrenia, indicating that D-Serine is intensely linked to memory and/or learning developments [32,33]. In addition, D-Serine depletion decreases the development of long-term potentiation (LTP) depending on the NMDA receptors, which is involved in the creation of memory [34]. Consistent with this, increased D-Serine levels may improve recognition and/or memory in rodents [35]. Accordingly, D-Serine creation might be a possible target to neutralize several brain disorders such as schizophrenia. D-Aspartate also seems to play an indispensable role in the neurotransmission system [36], and it is present in broad regions of the brain including the prefrontal cortex and/or hippocampus [37]. D-Aspartate has a considerable affinity at the L-Glutamate binding spot on the NMDA receptor [38] (Figure 1). D-Aspartate may increase during the development of the nervous system; however, the concentration of D-Aspartate radically decreases to a trace level by gestational week 41 and then continues at a very low level during the postnatal stages [39]. Degradation of D-Aspartate takes place via the D-aspartate oxidase (DDO) instead of the DAO (Figure 1), and the DDO is widely expressed in the brain [40]. Accordingly, the DDO is also considered an attractive therapeutic target [40]. D-Aspartate as well as D-Serine have been revealed to be involved in learning and/or memory [5,41]. 

## 3. Relationship between D-Amino Acids and Schizophrenia

Schizophrenia is a chronic neuropsychiatric disorder with abundant mortality, characterized by dissociations of ideas, identity, and emotions [42]. The underlying comprehensive causal mechanisms for schizophrenia remain unknown at present. Its clinical phenotype could be subdivided into positive symptoms such as hallucinations or delusions, and negative symptoms such as social withdrawal or impaired motivation, and/or those of cognitive impairment, which might result from the dysregulated neural network pathway in CNS [43]. The pathology of the disease appears to include complicated molecular abnormalities in the CNS. Hence, developmental dysfunction owing to the environmental and/or genetic factors in neurons might play a crucial role in the pathogenesis of schizophrenia [44]. Schizophrenia affects more than 20 million people worldwide [45]. Understanding the further molecular pathology of schizophrenia may lead to a superior diagnosis and/or treatment. 

Antagonists of NMDA receptors might aggravate patient symptoms with schizophrenia [46]. Since NMDA receptors’ hypofunction could also cause psychosis in humans, a better understanding of the NMDA receptors signaling mechanism may lead to a superior pharmacotherapy in schizophrenia [46]. Similarly, changed expressions of NMDA receptors involved in the metabolism of glutamate have been found in patients with schizophrenia [47]. Disturbances in the NMDA receptor-mediated synaptic transmission seem to be important factors. It has been reported that D-Serine and D-Aspartate are thought to play a role in NMDA-related synaptic plasticity with a potential involvement in schizophrenia [48]. In addition, D-Aspartate is noticed as being condensed in the synaptic vesicles of the axon-terminus in the developing brain, which suggests that its function is as a critical neurotransmitter in the growth of the CNS [49]. Furthermore, the NMDA receptor agonists may enhance anti-schizophrenic effects [50]. It has been shown that glutamatergic agents improve the negative symptoms of schizophrenia [50]. Consequently, DAO and serine racemase might be key enzymes for the association between D-amino acids and schizophrenia [51]. In fact, DAO-related genetic alterations have been related to the development of schizophrenia, and DAO inactivation produces behavioral effects with potential therapeutic benefits [52]. Consistently, the DAO gene has been shown to be a susceptibility gene for schizophrenia and its neurocognitive deficits, suggesting that DAO inactivation could result in showing anti-schizophrenic effects [51]. In addition, the stimulation of the DAO enzyme has shown enhanced symptoms in rodent models with schizophrenia, and an increased DAO activity seems most likely to impact the D-Serine metabolism [53]. Several studies have shown increased mRNA, protein, and/or enzymatic activity of DAO in post-mortem brain samples with schizophrenia [54]. A transcript of DAO has been detected in higher quantities in the schizophrenia-cerebellum [54]. Decreased D-Serine and/or the downregulation of NMDA receptors have resulted in a compromised synaptic plasticity, indicating a relation with the development of schizophrenia and deficits in learning and memory [55]. Decreased levels of D-Aspartate are also found in the brains of schizophrenic patients [56]. Remarkably, with schizophrenia, there has been a significant increase in D-Serine levels along with an improvement in clinical symptoms [57], which may be an effective antipsychotic treatment. Sodium benzoate, a DAO inhibitor, has also improved several symptoms in chronic schizophrenia patients [58]. Additionally, serine racemase knockout mice have shown an attenuation of seizure when compared with wild-type control mice, suggesting that serine racemase might be a target for the development of epileptic seizures’ therapeutic strategies [59]. A changed D-amino acid breakdown has also been associated with motor neuron degeneration as well as with schizophrenia. For example, decreased DAO is involved in motor neuron degeneration during senescence [59,60].

## 4. Involvement of Gut–Brain Axis via the Production of D-Amino Acids

It is well recognized that gut microbiota are the most significant regulator of the gut–brain axis [61]. The alteration of the composition of the gut microbiome could lead to schizophrenia. Dysfunctions in brain–gut communications might be related to certain gut inflammations. For example, it has been revealed that stress-related psychiatric symptoms such as irritable bowel syndrome show the substantial and physiological significance of the brain–gut axis [62,63]. It appears that the gut–brain interaction entails the direct excretion of some neuroactive matters. Intestinal microbiota may release several kinds of D-amino acids, which could be involved in the brain’s health [64]. In addition, bacterial glutamic acid racemases are the most abundant racemases, and they exist in peptidoglycan-containing bacteria in the gut microbiota [65]. D-amino acids are essential elements of peptidoglycans in the cell wall of bacteria [65]. Accordingly, the intestine in mammals is rich in free D-amino acids that might be derived from such bacteria within the gut microbiota [9]. On the other hand, the gut–brain axis could indicate a bidirectional communication between the nervous systems and intestinal functions in the microbiome [66]. It is probable that the D-amino acids’ metabolism in the brain might be modified by manipulating gut-microbiota bacterial communities [67]. Furthermore, it is possible that the gut microbiota could control brain function and/or affect brain development through epigenetic mechanisms [68]. Consistent with this, certain probiotics could be beneficial for the treatment of schizophrenia patients [69]. 

Dysfunction in the gut microbiota may be triggered by stressful situations, which could also affect a brain that is more susceptible to schizophrenia [70]. In addition, childhood trauma could modify the gut microbiota, which may also change the risk of schizophrenia [71,72]. Therefore, the association between gut microbiota and schizophrenia could be involved in schizophrenia pathogenesis [72]. Likewise, some reports have shown gut microbiota modifications in major depressive disorders [73]. Remarkably, depressive symptoms are also common features of schizophrenic animals and/or patients [74]. Increased therapeutic attention to mood symptoms would be desirable to support the prevention of schizophrenia. It has been shown that specific miRNA regulation in the prefrontal cortex could be affected by microbiota, which is required for the suitable control of miRNA in brains with anxiety behaviors [75]. In addition, an experiment with DAO knockout models has revealed certain differences in the gut microbiota composition, demonstrating a relationship between the activity of DAO in the gut and the composition of gut microbiota [76]. Although D-Serine and antipsychotics could not regulate Serine racemase and DAO protein levels, NMDA receptor neurotransmission could be regulated via the D-Serine availability in the brain [77]. Additionally, D-Serine derived from gut microbiota may also protect against acute kidney injury [67,78,79]. It has been reported that D-Serine is degraded by *Proteus mirabilis* [80]. On the other hand, *Enterococcus gallinarum* could have a Serine racemase activity that is able to racemize Serine more efficiently than Alanine [81,82]. Serine racemases are distributed widely in various bacteria including *Escherichia coli* [83]. Additionally, the expression of DAO has been shown in the yeast *Schizosaccharomyces pombe* [84].

## 5. D-amino Acids as a Useful Biomarker

Effective biomarkers should be used in schizophrenia patients, as their usage might help in the prediction of the disease, prognosis, therapy response, and/or regulation of adverse effects in treatment [85]. Therefore, it is significant to investigate valuable biomarkers demonstrating the current pathology of schizophrenia. These valuable biomarkers may be divided into peripheral and brain/CNS biomarkers. In particular, the blood plasma-based biomarker is really useful to reveal some pathological progressions in the brain [86]. Several alterations in epigenetic and/or in proteomic markers have also been detected in the periphery as well as in the brain/CNS [87]. 

D-amino acids are known as physiologically active substances that are useful biomarkers for several brain disorders in mammals [88]. The D-Serine/L-Serine ratio in the CSF has been reduced in the postmortem brain of schizophrenic patients, although the levels of L-serine and L-glutamate in the CSF are unaffected [89]. Similarly, a meaningfully reduced D-Serine/total-Serine ratio in the CSF of schizophrenic patients has also been shown [90]. As mentioned formerly, D-Serine complemental treatment could improve positive, negative, and cognitive symptoms in patients with schizophrenia [91]. In relation to this, G72/G30, a modulator of DAO, has been implicated in schizophrenia [92]. In fact, plasma G72/G30 levels were also found to be significantly higher in schizophrenia patients than in healthy controls [92]. Afterwards, a number of studies have reported evidence of the relationship between the G72/G30 and schizophrenia [93,94]. Furthermore, it has been revealed that DAO and G72/G30 are implicated as key proteins in the NMDA receptor signaling pathway for schizophrenia [95]. Furthermore, plasma DAO levels have also increased in post-stroke dementia patients, suggesting an effective biomarker for the diagnosis of dementia [96]. The peripheral DAO levels may increase with age-related cognitive decline, supporting the hypofunction of the NMDA receptor in the dementia brain [97]. Imminent molecular work is required to further validate the contribution of G72/G30 and DAO to the pathogenesis of schizophrenia. 

**Figure 2:**
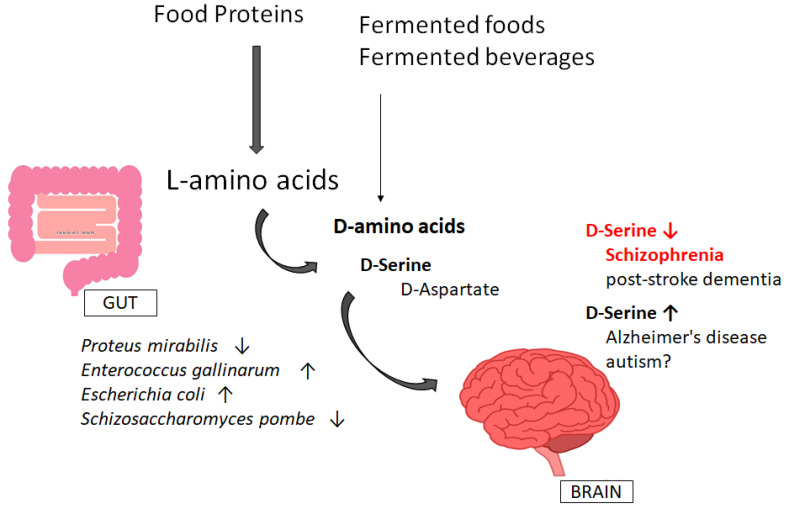
Gut microbiota might contribute to the creation or the destruction of the D-amino acids, which could play key roles in the pathological processes of psychiatric diseases. The bacteria shown here are instances that are involved in the increase (↑) or decrease (↓) of certain D-amino acid levels. Consequently, decreased levels of D-Serine may be associated with schizophrenia, whereas increased D-Serine levels might be found in Alzheimer′s disease. As a remark, critical events such as ROS production, cytokine induction, and immune activation have been omitted for simplicity.

In contrast, a study has proposed that increased levels of D-Serine could predict worse memory-dwindling symptoms [97]. The levels of D-Serine in both plasma and CSF have been found to be considerably higher in patients with Alzheimer′s disease [97,98]. Therefore, D-Serine and the D-Serine/total serine ratio have also been suggested as biomarkers of Alzheimer’s disease progression [99]. In addition, the racemization of Aspartate at position 23 of the beta protein in the amyloid deposit has enhanced its aggregation and/or fibril formation in the Alzheimer′s disease brain [100]. Additionally, D-Aspartate and D-Serine are key neuromodulators of glutamatergic synaptic transmission in autism spectrum disorders (ASD) [101]. To summarize, decreased D-Serine levels have been reported in schizophrenia patients, whereas increased levels of D-Serine have usually been detected in Alzheimer’s disease (Figure 2).

## 6. Perspectives

D-amino acids seem to be indispensable signaling molecules in neural systems. Furthermore, the recognition of the D-amino acids by the immune system might modulate immunity signals [102]. The regulation of D-amino acids also has important implications at microbe-to-host crossing points [102]. It has been shown that prenatal immune activation by infection might be an environmental risk factor for schizophrenia via the NMDA receptor-mediated synaptic dysfunction [103]. Therefore, developments to identify trace D-amino acids with a high sensitivity would facilitate the progression of the D-amino acids field [104]. An adjusted D-amino acids detection supporting the examination of clinical specimens would also assist future studies in this field. In addition, an advanced tool with superior biosensors for D-amino acids as a biomarker will accelerate imminent research that is directed at discovering the neurological role of D-amino acids [105]. The roles of D-amino acids and gut microbiota in the molecular pathogenesis of schizophrenia could be an exciting subject to explore in the future. Therefore, additional research into the impact of D-amino acids on neuronal roles is extremely anticipated at present. Linking biomarkers and drug development for schizophrenia is also critical in forthcoming studies. In particular, the involvement of both the diagnosis and treatment of schizophrenia might result in further beneficial potential toward achieving this goal. For example, several clinical studies with promising results suggest that D-Serine could actually be effective in schizophrenia patients [106]. D-Serine administered in arrangement with traditional antipsychotics might be more beneficial in treating patients with schizophrenia. A key component in the pathological mechanism for schizophrenia may be the dysfunction of the NMDA receptor. Considering the fact that D-Serine, D-Glutamate, and D-Alanine may play characteristic roles in Alzheimer′s disease [107], NMDA receptor modulators could also be potential therapeutic drugs in schizophrenia [108]. However, the high doses of D-Serine may cause peripheral neuropathic pain [109]. Accordingly, it would be worth precisely checking the changes in D-amino acid levels in patients.

## Data Availability

The datasets used and/or analyzed in the current study are available from the corresponding author on reasonable request.

## Abbreviations

ALS	amyotrophic lateral sclerosis
ASD	autism spectrum disorders
ATP	adenosine triphosphate
CNS	central nervous system
CSF	cerebrospinal fluid
DAO	D-amino acids oxidase
DAOA	DAO activator
DDO	D-Aspartate oxidase
DNA	deoxyribonucleic acid
LTP	long-term potentiation
miRNA	microRNA
mTOR	mammalian target of rapamycin
NMDA	N-methyl-D-aspartate
ROS	reactive oxygen species
SOD	superoxide dismutase
